# PIPS: Pathogenicity Island Prediction Software

**DOI:** 10.1371/journal.pone.0030848

**Published:** 2012-02-15

**Authors:** Siomar C. Soares, Vinícius A. C. Abreu, Rommel T. J. Ramos, Louise Cerdeira, Artur Silva, Jan Baumbach, Eva Trost, Andreas Tauch, Raphael Hirata, Ana L. Mattos-Guaraldi, Anderson Miyoshi, Vasco Azevedo

**Affiliations:** 1 Department of General Biology, Federal University of Minas Gerais, Belo Horizonte, Minas Gerais, Brazil; 2 Department of Biochemistry and Immunology, Federal University of Minas Gerais, Belo Horizonte, Minas Gerais, Brazil; 3 Department of Genetics, Federal University of Pará, Belém, Pará, Brazil; 4 Department of Computer Science, Max-Planck-Institut für Informatik, Saarbrücken, Saarland, Germany; 5 Center for Biotechnology, Bielefeld University, Bielefeld, Nordrhein-Westfalen, Germany; 6 Microbiology and Immunology Discipline, Medical Sciences Faculty, State University of Rio de Janeiro, Rio de Janeiro, Brazil; St. Petersburg Pasteur Institute, Russian Federation

## Abstract

The adaptability of pathogenic bacteria to hosts is influenced by the genomic plasticity of the bacteria, which can be increased by such mechanisms as horizontal gene transfer. Pathogenicity islands play a major role in this type of gene transfer because they are large, horizontally acquired regions that harbor clusters of virulence genes that mediate the adhesion, colonization, invasion, immune system evasion, and toxigenic properties of the acceptor organism. Currently, pathogenicity islands are mainly identified *in silico* based on various characteristic features: (1) deviations in codon usage, G+C content or dinucleotide frequency and (2) insertion sequences and/or tRNA genetic flanking regions together with transposase coding genes. Several computational techniques for identifying pathogenicity islands exist. However, most of these techniques are only directed at the detection of horizontally transferred genes and/or the absence of certain genomic regions of the pathogenic bacterium in closely related non-pathogenic species. Here, we present a novel software suite designed for the prediction of pathogenicity islands (pathogenicity island prediction software, or PIPS). In contrast to other existing tools, our approach is capable of utilizing multiple features for pathogenicity island detection in an integrative manner. We show that PIPS provides better accuracy than other available software packages. As an example, we used PIPS to study the veterinary pathogen *Corynebacterium pseudotuberculosis*, in which we identified seven putative pathogenicity islands.

## Introduction

Bacteria are the most abundant and diverse organisms on Earth [1]. This diversity is mainly the result of the remarkable genomic plasticity of bacteria, which allows bacteria to adapt to a wide range of environments, enhancing their pathogenic potential [2], [3]. Various mechanisms can promote genome plasticity, including point mutations, gene conversion, chromosome rearrangements (inversions and translocations), deletions, and the acquisition of DNA from other cells through horizontal gene transfer (HGT). Those mobile elements can be acquired via plasmids, bacteriophages, transposons, insertion sequences and genomic islands (GEIs) [4].

GEIs play a major role in the fast and dramatic adaptation of species phenotypes to different environments by carrying clusters of genes that can cooperate to confer a cell with novel and useful phenotypes, such as the ability to survive inside a host. GEIs are large genomic regions that present deviations in codon usage, G+C content or dinucleotide frequency compared to other parts of the organism's genome; these characteristics are hallmarks of chromosome regions that were acquired horizontally from other species in a single block. GEIs are often flanked by insertion sequences or tRNA genes and transposase coding genes; these segments are responsible for the genomic incorporation of alien DNA obtained through transformation, conjugation or bacteriophage infection [5].

### Horizontally acquired genes

GEIs acquired by transposase-mediated insertion have inverted repeats (IR) or insertion sequences (IS) in their flanking regions and often harbor tRNA coding sequences [6]. Genes coding for tRNA and tmRNA (hereafter tRNA genes) are “hot spots” for the insertion of genetic elements; they possess a 3′-terminal sequence that is recognized by integrases and are frequently found in *selC* and *leuX* tRNA genes (selenocysteine and leucine, respectively) [6], [7].

The identification of horizontally acquired regions is usually based on the detection of a chromosome region's G+C content and codon usage that differs from that found in the rest of the genome. Clusters of horizontally acquired genes may have a skewed G+C content and codon usage, reflecting a distinct genomic signature from a donor organism [8]. Although these G+C content-skewed regions within an acceptor organism genome remain functional to some extent, there is selective pressure for the acquired region to adapt its codon usage to that of the acceptor organism to enhance expression. This adaptation in codon usage is driven by selective forces, such as codon/anticodon linkage and a greater frequency of a certain codon for the tRNA gene [9]. Codon usage bias in bacteria is closely related to base composition, and the adoption of preferential G+C- or A+T-rich codons may lead to a similar G+C content of genes throughout the genome [10]. Given the high density of coding regions in prokaryotic genomes, codon usage adaptation, in addition to point mutations and other evolutionary forces, can lead to homogeneity in the base composition of bacteria. Consequently, the identification of mobile genomic regions based solely on their discrepant genomic signature is usually only possible for regions that were recently acquired from distant organisms [11], [12].

In addition to the aforementioned features, Hsiao *et al.* [13] demonstrated that GEIs have a high frequency of hypothetical proteins (putative proteins with unknown function) when compared to the rest of the genome. These investigators indicated that this higher frequency could result from gene acquisition from organisms that have not yet been sequenced, including non-culturable bacteria.

### Virulence factors and pathogenicity islands

GEIs may carry a number of coding regions that are useful for a cell. The GEIs that carry gene coding for virulence factors are collectively known as pathogenicity islands (PAIs). PAIs are characterized by the high frequency of genes that code for factors that enable or enhance the parasitic growth of the microorganism within a host [14]. Virulence factors mediate adhesion, colonization, invasion, immune system evasion and toxigenesis, which are necessary for infection [15].

Hacker *et al.* [5] first described PAIs after observing the loss of virulence of pathogenic varieties of *Escherichia coli* through deletions of hemolysin and fimbrial adhesin genes. They demonstrated that these genes are located in the same chromosomal region and can be removed by deletion events, both *in vitro* and *in vivo*. PAI identification using traditional molecular biology techniques without genomic information services is laborious and time-consuming because of the need for phenotypic analyses of the strains and the delimitation of the target genes. Additionally, PAIs often present variable stability, mosaic structure and uncharacterized genes.

### *In silico* analysis of pathogenicity islands

PAI analysis is becoming more feasible with the increasing number of sequenced prokaryotic genomes and the development of new bioinformatics methods that can assemble data retrieved from next-generation sequencers (NGS). NGS plataforms have the potential to increase the number of completed genome projects orders of magnitude more rapidly than the earlier Sanger method and at a small fraction of the cost. Consequently, the need for the development of genomic data retrieval softwares is increasing. Several computational programs have been specifically designed for spotting PAIs and other HGTs. However, most of the programs use criteria that are not sufficiently stringent to provide useable sensitivity and specificity. Overall, existing software only screens for horizontal gene transfer, through G+C content or dinucleotide deviations (e.g., wavelet analysis of the G+C content, cumulative GC profile, δ_P_-web, IVOM, IslandPath and PAI-IDA) [16]–[23] and codon usage deviation (SIGI-HMM and PAI-IDA) [16], [24] or for the absence of elements of the putative PAI in non-pathogenic species (IslandPath, Islander, IslandPick and tRNAcc) [7], [8], [20], [25], which may result in the detection of false-positive PAIs [8], [26]. Pundhir *et al.* [27] affirm that “Although efficient in the detection of GIs, these tools give much false positive results for PAIs. This is because a region showing distinct nucleotide content may be alien to the host genome but may not necessarily be involved in Pathogenicity”. Therefore, these tools may detect a metabolic island, a GEI associated with secondary metabolite biosynthesis, as a false-positive PAI if it exhibits all of the PAI features except for the virulence factors. Finally, some PAIs may exhibit deviations only in the G+C content or codon usage, demonstrating the importance of using more than one software system in a multi-pronged approach.

Two currently available PAI detection programs use a multi-pronged strategy for the detection of PAIs, accounting for several characteristics of the genome. One of these programs, PredictBias, identifies PAIs by its genomic signature, its absence in taxonomically related organisms and the presence of genes coding for virulence factors, classifying them as either biased-composition PAIs if they present horizontal transfer characteristics or unbiased-composition PAIs otherwise [27]. Another program, IslandViewer, performs a combined analysis using three other programs: ColomboSIGI-HMM, based on codon usage analysis of each coding sequence (CDS) of the genome; IslandPick, which characterizes PAIs by their absence in phylogenetically closely related organisms; and IslandPath-DIMOB, which finds regions that have dinucleotide content deviation and harbor genes related to mobility [8], [28], [29].

Although PredictBias and IslandViewer are robust programs that use multi-pronged strategies, they have some restrictions. For example, PredictBias can only be used in a web-based interface; the genome sequence must be sent to the server to be analyzed. A web-based interface can be a limitation, such as when the genome sequence is not yet published and, thus, the data cannot be sent to third parties. Island Viewer, on the other hand, includes a source code for installation on a personal server. However, IslandPick, one of the programs that Island Viewer requires, is strongly dependent on an in-house MySQL database of all published bacterial genomes, which make its use very time-consuming. Moreover, this program requires a very fast server with an unconventional configuration.

Our main goal in this work was to develop new software to predict PAIs with more efficiently than currently available software and to make the software easier to install on a personal computer. Our software, PIPS (pathogenicity island prediction software), predicts PAIs using a novel and more complete approach based on the detection of multiple PAI features: atypical G+C content, codon usage deviation, virulence factors, hypothetical proteins, transposases, flanking tRNA and its absence in non-pathogenic organisms.

In the next sections, we describe the implementation of this software, which is used with several other tools. Model organisms of the genera *Corynebacterium* and *Escherichia* were used in the validation process. The results and discussion section includes data derived from the analyses of *Corynebacterium diphtheriae* and *Escherichia coli* that validate and prove the superior efficiency of this program over other multi-pronged tools. We also performed a case study on *Corynebacterium pseudotuberculosis* that demonstrates the importance of examining various PAI features along with comparisons of PAIs between closely related species.

## Materials and Methods

**Figure 1:**
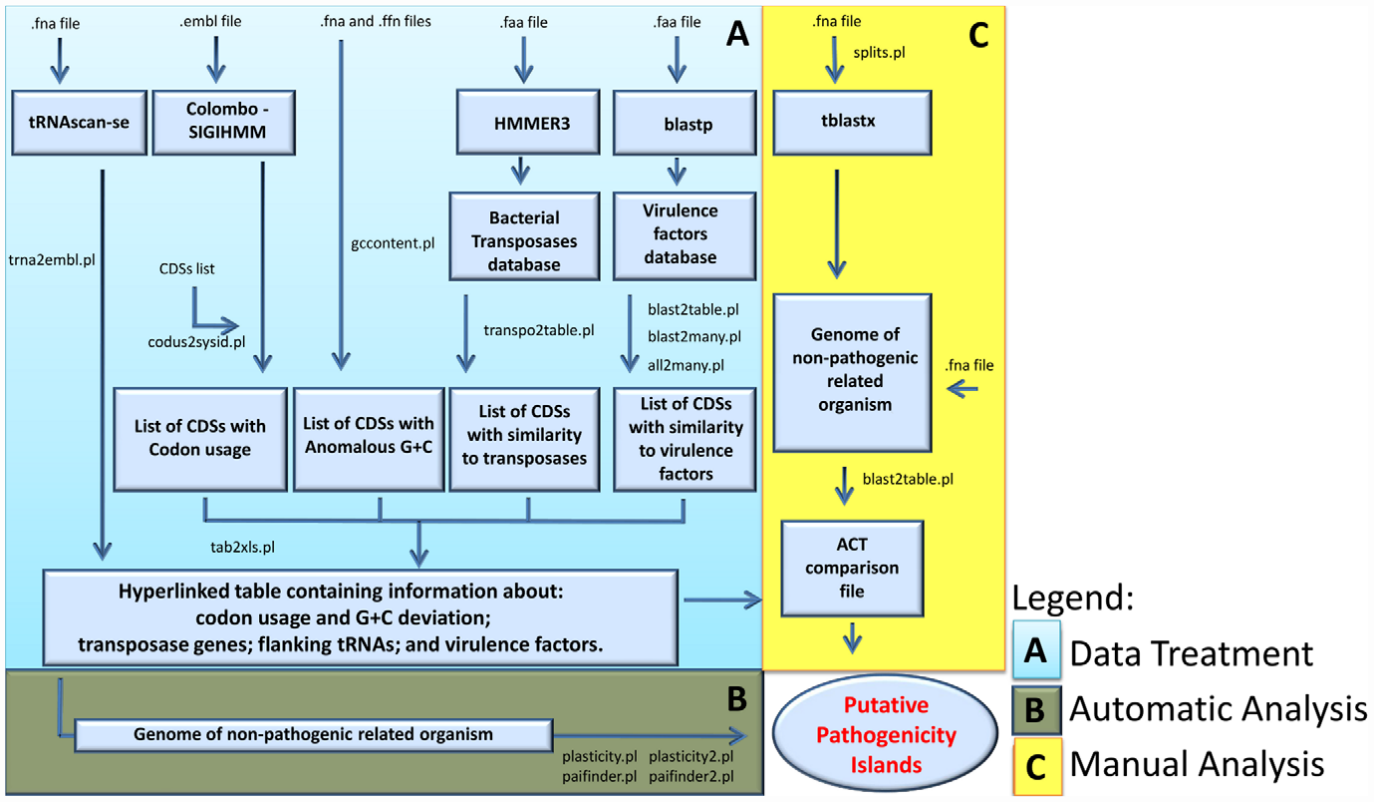
Flowchart presenting each PAI analysis step performed by PIPS. The procedure is divided into the following steps: (A) data treatment; (B) automatic analyses; and (C) manual analyses.

The steps that are required to use PIPS and the necessary input information are represented in the flowchart in Figure 1.

### Genomic signature

Putatively acquired regions are identified based on the analysis of G+C content and codon usage patterns, as described below.

#### Codon usage deviation

The Colombo SIGI-HMM software was used to predict acquired genes and their putative origins based on taxon-specific differences in codon usage [29]. This software analyzes sequences of predicted proteins of an .embl input file using a hidden Markov model (HMM). This method considers a pattern of observations issued from a hidden Markov chain structure. Additionally, Colombo SIGI-HMM allows the parameter sensitivity to be configured. We pre-configured the parameter sensitivity to 95% to detect any minor anomalies in codon usage because the data are subjected to other major analyses at later stages.

#### G+C deviation

The Artemis software includes a tool that detects regions with atypical G+C content. This tool calculates the mean G+C content of the genome along with its standard deviation and uses 2.5 standard deviations (SD) as a boundary limit (cutoff) to predict regions with atypical G+C content [30]. The high accuracy of this tool is due to its 1,000-base window size, which identifies even intergenic regions. However, the standard deviation boundary cannot be configured in this program. The base composition of the genome and its coding sequences (CDSs) were analyzed with a Perl script, using input files in .fna and .ffn formats. The script also analyzes the G+C content of the genome and each CDS using 1.5 SD as a boundary to identify putatively acquired regions, as described by Jain *et al.* [31].

**Figure 2:**
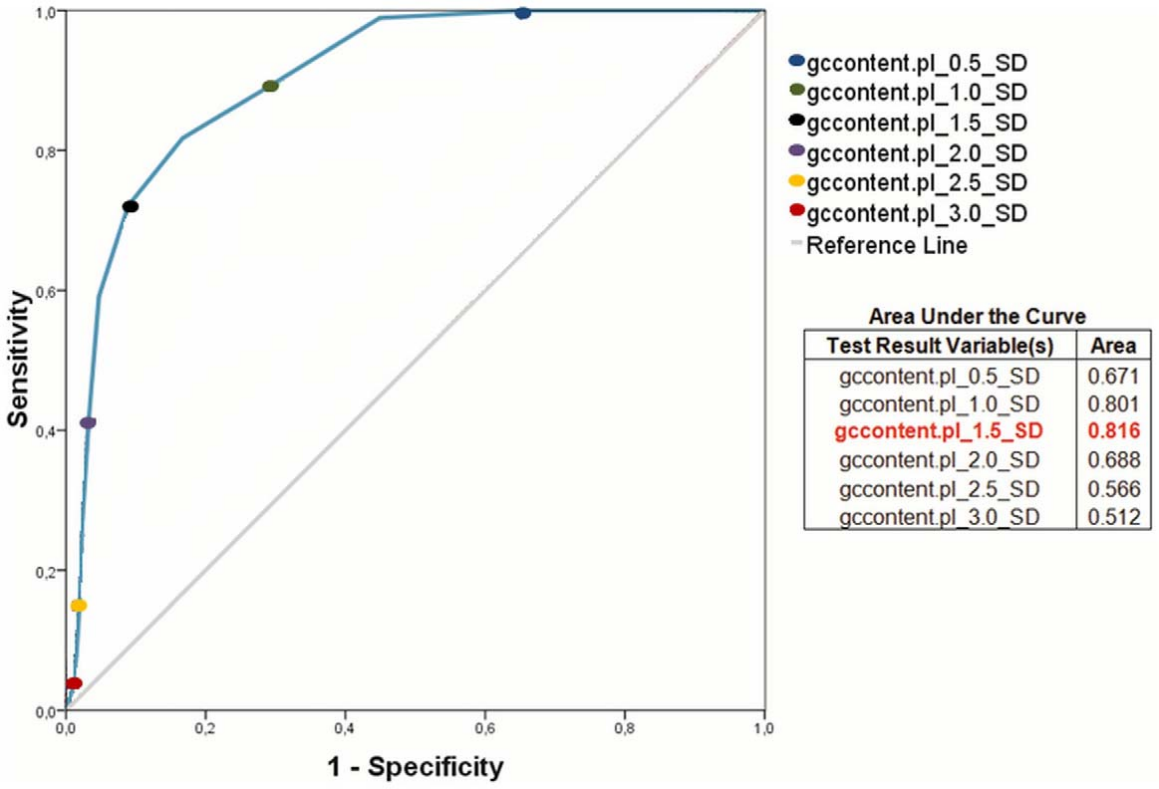
ROC curve showing the sensitivity and specificity of the Perl script for the identification of regions with GC content deviation. Y-axis: sensitivity; X-axis: 100-specificity. The higher the accuracy is, the closer the curve is to the upper-left corner.

To validate the script, the complete *C. diphtheriae* genome was analyzed using Artemis to generate a positive dataset of all genome CDSs with atypical G+C; the sensitivity and specificity of the method were calculated with configurations varying from 0.1 to 3.0 SD. These data were plotted and analyzed in a receiver operating characteristic (ROC) curve (Figure 2) [32].

Based on the ROC curve, the boundary is located between 1.0 and 1.5 SD. The area under the curve (AUC) was then analyzed to determine the most precise value, i.e., the value that gives the largest AUC (Figure 2) [32], which corresponds to the output data generated by the script with a 1.5 SD boundary configuration.

### Transposases

Putative transposase genes are identified by PIPS, which uses HMMER3 [33] to search a bacterial transposase protein database that was retrieved from the Pfam protein families database [34]. The HMMsearch only considers alignments with an e-value of 1e-5 to avoid erroneous alignments that could result in false-positive prediction of transposase genes. A Perl script was created to process the HMMER3 output file and generate a list of putative transposases.

### Virulence factors

Virulence genes are identified using BLASTP (BLAST-NCBI [35]) searches with an e-value of 1e-5 against a virulence factor database, mVIRdb. This database contains proteins from eight sources, including toxin, virulence factor and antibiotic resistance gene sequences [36].

### Hypothetical proteins

The term “hypothetical protein” is used to identify putative coding sequences without significant matches against non-redundant protein and protein domain databases during genome annotation. Data from annotation in the genome .embl file are used to identify hypothetical proteins. Alternatively, automatic annotation of a whole genome nucleotide file can be processed on our website using an annotation tool (Annotatiohmm). Annotatiohmm is an additional software system that is specifically designed to predict ORFs using the software genemark [37], based on a closely related species HMM profile. After the prediction, it performs HMM searches in the Pfam protein families database to create an .embl file, which can be used by PIPS [33], [34].

### Transfer RNAs

Transfer RNA genes are identified by the software tRNAscan-SE [38], and the output file is parsed by a Perl script to generate a file that can be used in Artemis and ACT (Artemis comparison tool) software to identify flanking tRNAs.

### Genomic plasticity

Genomic plasticity analyses are performed using the premise that most pathogenicity islands are absent in non-pathogenic organisms of the same genus or other related species [4]. PIPS analyses may also be performed with a closely related pathogenic organism. However, the pathogenicity islands shared by the two organisms will not be detected during the identification process. In addition, it may erroneously identify other classes of GEIs (e.g., resistance islands and metabolic islands) as PAIs. Therefore, the use and careful choice of the non-pathogenic species is crucial.

PIPS performs two different analyses to identify regions with genomic plasticity. First, an automatic analysis generates a list of putative pathogenicity islands. Second, it creates files that can be manually analyzed to complement and curate the automatic analysis.

#### Automatic analysis

After the identification of genes that are related to virulence and CDSs presenting characteristics that suggest horizontal transfer, PIPS performs a protein similarity search using BLASTP with the pathogenic bacterium (query) against a non-pathogenic species (subject). The input file in this step contains the predicted protein sequences from the two genomes, and the BLASTP is performed with an e-value of 1e-5. The blastp output file is parsed by Perl scripts that find regions of the non-pathogenic bacterium (subject) that are absent in the pathogenic bacterium (query). Finally, the CDSs are clustered in major regions using their genome coordinates and are identified as “putative pathogenicity islands” based on the finding of virulence factors and characteristics that indicate horizontal transfer, i.e., G+C content deviation or codon usage deviation at higher frequencies than found in the whole genome sequence.

#### Manual analysis

A second protein search is performed using tblastx against the non-pathogenic species with an e-value of 1e-5. The output file is parsed by a Perl script, generating a comparison file that can be used in the ACT software. This tool permits the visualization of protein similarity areas and insertion, deletion, translocation and inversion regions [39].

### The Corynebacterium genus

*Corynebacterium diphtheriae* strain NCTC 13129 [GenBank: BX248353] – This microorganism is the etiological agent of diphtheria, an infectious disease of the upper respiratory tract, which has been largely controlled by widespread vaccination. Diphtheria has re-emerged in some regions, however, especially in Europe, causing considerable mortality because of the appearance of new biotypes and inadequate vaccination [40].

*C. diphtheriae* was chosen to validate PIPS because it is a pathogenic species with 13 putative PAIs that is closely related to *C. pseudotuberculosis*. These 13 PAIs were identified by performing analyses based on the following: anomalies in nucleotide composition (e.g., G+C content, GC skew and/or dinucleotide frequency); their absence in *Corynebacterium glutamicum* and *Corynebacterium efficiens*; flanking tRNAs; and the presence of genes encoding virulence factors, such as fimbrial and fimbria-related genes, iron-uptake systems, a potential siderophore biosynthesis system, a lantibiotic biosynthesis system, exported proteins, two-component-system proteins, insertion sequence transposases and the *tox* gene, which is located in a corynephage-acquired region and is responsible for the pathognomonic symptoms of diphtheria [41].

*C. glutamicum* strain ATCC 13032 [GenBank: BX927147] was chosen for the comparison analyses, which is non-pathogenic and of biotechnological interest, being widely used for the industrial production of amino acids such as L-glutamic acid and L-lysine [42].

*C. pseudotuberculosis* strains 1002 [GenBank: CP001809] and C231 [GenBank: CP001829] were chosen to test PIPS after validation, both of which are facultative intracellular pathogens. This species is the etiological agent of the globally distributed disease known as caseous lymphadenitis (CLA), which mainly affects small ruminants. However, this bacterial species can affect a wide range of host species, causing different diseases. *C. pseudotuberculosis* is less well studied than *C. diphtheriae*. The virulence factors of *C. pseudotuberculosis* that lead to CLA have not yet been exhaustively characterized, making studies concerning PAIs in this species invaluable [43].

### The Escherichia coli species

Among the *E. coli* species, we chose the uropathogenic *E. coli* (*UPEC*) strain *CFT073* [GenBank: AE014075], a pyelonephritogenic *UPEC* isolate that has a wide range of putative and known virulence genes that are responsible for survival in the host. The *UPEC* strains deserve great attention because they are responsible for up to 90% of uncomplicated urinary tract infections. In addition, using comparative genomic hybridization analysis and combining genomics, bioinformatics, and microarray technologies, 13 pathogenicity islands larger than 30 kb have already been described in *E. coli* strain CFT073 [44].

*Escherichia coli* strain *K-12*, substrain *MG1655* [GenBank: U00096], was chosen for the genomic plasticity comparison with the *UPEC* strain *CFT073* because it is the best-studied non-pathogenic strain of this species. In addition, the genomic sequence of this strain undergoes constant curation and updating, reducing erroneous annotations [45], [46].

## Results and Discussion

### Software validation using *C. diphtheriae* PAIs

A genomic region was identified as a putative PAI of *C. diphtheriae* (PICD) when it had the following properties. First, it presented most of the PAI features in *C. diphtheriae* (e.g., higher concentration inside the genomic region than in the whole genome of virulence factors and/or hypothetical proteins and CDSs with codon usage deviation and/or atypical G+C content). Second, it was absent in *C. glutamicum*. PIPS found 12 of the 13 *C. diphtheriae* PAIs; except for *C. diphtheriae* PICDs 10 and 13, all of the islands were 1–7 CDSs larger than the published sequences (Figure S1).

### Comparison between PIPS and other programs

To compare the efficiency of PIPS in identifying PAIs with the results of other available programs, we analyzed the sensitivity and specificity using published data, with *C. diphtheriae* PAIs as a positive dataset (Table 1). For this task, each CDS in a genome was labeled as “positive” when it was harbored by a PAI and “negative” otherwise. For more detailed information concerning the composition of PAIs predicted by the programs, see Table S1.

**Table 1:** Comparison between the software used to identify pathogenicity islands in the *C. diphtheriae* strain *NCTC 13129*.

Software	Sensitivity (%)	Specificity(%)	Accuracy(%)
IslandPath_DIMOB	13.6	98.3	89.2
IslandPick	65.2	81.9	80.1
SIGI_HMM	14.0	94.9	86.2
IslandViewer	74.4	76.4	76.2
PredictBias_GEI	30.8	84.4	78.6
PredictBias_PAI	2.4	88.7	79.4
PIPS_Auto	86.4	85.0	85.1
PIPS_Manual	96.8	87.1	88.1

PredictBias showed good specificity (88.7%), at the cost of sensitivity (2.4%), when using only predicted PAIs (PredictBias_PAI) as a positive dataset for the test (Table 1). The sensitivity was higher (30.8%) when GEIs identified by the program (Table 1) were used as a positive dataset (PredictBias). The classification errors may be a consequence of the virulence factor database used by the program. The database was created using an NCBI search with the following keywords: ‘Virulence’, ‘Adhesin’, ‘Siderophore’, ‘Invasin’, ‘Endotoxin’ and ‘Exotoxin’ [36]. The size of the database is a determining factor in discerning PAIs from GEIs. The larger the database is, the higher the probability of correct classification of a gene as a virulence factor and, consequently, the higher the probability of correct PAI identification.

IslandViewer identified 10 *C. diphtheriae* PAIs; however, their sizes varied from those of the published PAIs. Two of the three programs used in IslandViewer, IslandPath-DIMOB and Colombo/SIGI-HMM, had low sensitivity for PAI prediction (13.6% and 14%, respectively). However, the poor performance of Colombo/SIGI-HMM mainly results from the high stringency of its parameters. In our case, setting the program's “sensitivity” parameter to 95% resulted in higher sensitivity and proved to be an efficient approach for the identification of regions with codon usage deviation.

IslandPick had a higher sensitivity (65.2%) than the other programs used in IslandViewer (Table 1). This software performs analyses that are based on the premise that PAIs are absent in related non-pathogenic organisms. The superior performance of this strategy corroborates the importance of genomic comparisons between the bacterium to be analyzed and a non-pathogenic strain or species of the same genus. Finally, the programs IslandPick, IslandPath-DIMOB and Colombo/SIGI-HMM, when combined in IslandViewer, gave a higher sensitivity for predicting PAIs (74.4%) than when used alone (65.2%, 13.6% and 14.0%, respectively), which demonstrates the importance of a combined analysis instead solely analyzing a single PAI feature.

PIPS correctly identified 12 of the 13 PAIs. Based on *C. diphtheriae* genomic annotation, the only PAI that was not identified by PIPS, PICD 5 of *C. diphtheriae*, has an atypical G+C content of 52.2%. However, when a boundary value of 1.5 standard deviations was used to identify atypical G+C content, we found reference values that varied from 45.95 to 60.04%. In addition, when using Artemis, the annotation tool did not indicate any atypical G+C in this PAI, which is in agreement with PIPS. Moreover, except for its absence in *C. glutamicum*, PICD 5 of *C. diphtheriae* did not show any other PAI feature. Additionally, the IslandViewer and PredictBias results also indicate that the classification of PICD 5 of *C. diphtheriae* as a PAI is erroneous.

Finally, automatic analysis using PIPS gave better performance than the previously available techniques (86.4% sensitivity, 85.0% specificity). However, manual analysis of PIPS results in improved identification of the PAIs (96.8% sensitivity, 87.1 specificity), showing the importance of manual curation of the data based on biological knowledge.

### Identification of the well-studied pathogenicity islands of the uropathogenic *E. coli* strain *CFT 073*

After the validation of PIPS with a Gram-positive bacterium, we analyzed the *UPEC* strain CFT073 to determine how well PIPS performs with a Gram-negative bacterium. Gram-negative bacteria are important in this context because their PAIs tend to present all of the PAI features concurrently; additionally, *E. coli* PAIs have been extensively described in the literature [5], [7], [44], [47]–[51]. The *UPEC* strain CFT073 was chosen because it possesses several known PAIs. We used 13 PAIs described by Lloyd *et al.* [44] as our gold standard and compared the accuracy of PIPS with IslandViewer and PredictBias, as we had performed with *C. diphtheriae*. The *E. coli* strain *K-12* was used as the non-pathogenic closely related organism for validation in this step. The sensitivity and specificity of the methods are shown in Table 2.

**Table 2:** Comparison between the software used to identify pathogenicity islands in the uropathogenic *E. coli* strain *CFT 073*.

Software	Sensitivity (%)	Specificity(%)	Accuracy(%)
IslandPath_DIMOB	44.5	99.3	90.2
IslandPick	7.5	99.7	84.5
SIGI_HMM	21.9	96.9	84.5
IslandViewer	55.8	96.2	89.5
PredictBias_GEI	60.0	93.7	88.1
PredictBias_PAI	39.2	96.2	86.8
PIPS_Auto	94.8	93.7	93.9

The specificity achieved by the other methods (93.7–99.3%) was greater than that of PIPS (93.7%), although PIPS had a much higher sensitivity (94.8%) than the other methods (7.5–60%). This reduced specificity may result from novel pathogenicity islands that were not previously identified rather than false-positive results. In addition, the higher accuracy of PIPS (93.9%) when compared to the other methods (84.5–90.2%) supports our previous conclusion that PIPS gives the best performance when identifying true positive and true negative CDSs, based on the analysis of PAIs of the *UPEC* strain *CFT073*.

### Case study: C. pseudotuberculosis

After validating PIPS, we identified putative PAIs of *C. pseudotuberculosis*. The underlying properties (i.e., codon usage, G+C content, virulence factors and hypothetical proteins) of the *C. pseudotuberculosis* (PICPs) and *C. diphtheriae* (PICDs) PAIs are given in Table 3. For further details, please refer to Figure S2.

**Table 3:** Percentage of PAI features along the genome and the pathogenicity islands of *C. pseudotuberculosis* and *C. diphtheriae*.

	Codon usage deviation (%)	GC content deviation (%)	Virulence factors (%)	Hypothetical proteins (%)
*C. diphtheriae* NCTC 13129 PICDs	45.20	20.80	18.40	39.20
*C. diphtheriae* NCTC 13129 genome	26.89	9.52	17.45	27.19
*C. pseudotuberculosis* 1002 PICPs	14.79	23.08	30.77	31.95
*C. pseudotuberculosis* 1002 genome	3.52	11.65	17.27	31.95
*C. pseudotuberculosis* C231 PICPs	19.62	20.25	32.91	31.65
*C. pseudotuberculosis* C231 genome	3.80	10.76	17.77	31.64

#### G+C content

*C. pseudotuberculosis* PICPs had similar frequencies of CDSs with G+C content deviations to those identified in *C. diphtheriae* PICDs. Compared to the frequency in their respective genomes, the frequency of CDSs with G+C content deviation in *C. pseudotuberculosis* PICPs and *C. diphtheriae* PICDs was approximately doubled.

#### Codon usage

The frequency of CDSs with codon usage deviation was found to be higher in the *C. diphtheriae* PICDs than in the *C. pseudotuberculosis* PICPs, reflecting the patterns found in the genomes of *C. diphtheriae* and *C. pseudotuberculosis* (Table 3). However, the frequency of CDSs with codon usage deviation in *C. pseudotuberculosis* PICPs, although lower than the frequency in *C. diphtheriae* PICDs, was three times that in the *C. pseudotuberculosis* genome (Table 3). In PICDs, the frequency of this feature was twice that in the whole genome.

#### Virulence factors

The frequency of virulence factors in *C. pseudotuberculosis* PICPs is approximately twice that in other parts of the *C. pseudotuberculosis* genome, in contrast to findings in *C. diphtheriae* PICDs (Table 3). When looking at PAIs separately, the frequencies of virulence factors in *C. pseudotuberculosis* PICPs were also higher than in *C. diphtheriae* PICDs; however, *C. diphtheriae* PICDs had higher frequencies of hypothetical proteins, i.e., putative proteins without significant similarity to any previously described protein (Table 3). These proteins may have an unknown role in pathogenicity, possibly explaining the low frequencies of the possible virulence factors found in these regions.

### Frequencies of the features in each *C. pseudotuberculosis* PICP

The properties that were analyzed in a global genomic view in the previous section (i.e., codon usage, G+C content, virulence factors and hypothetical proteins) were assessed for each *C. pseudotuberculosis* PICP to compare their contributions to the classification. To plot this graph, we used the frequency, in percent, of the CDSs, presenting the chosen feature in the *C. pseudotuberculosis* PICP relative to the total number of CDSs in the same PICP.

**Figure 3:**
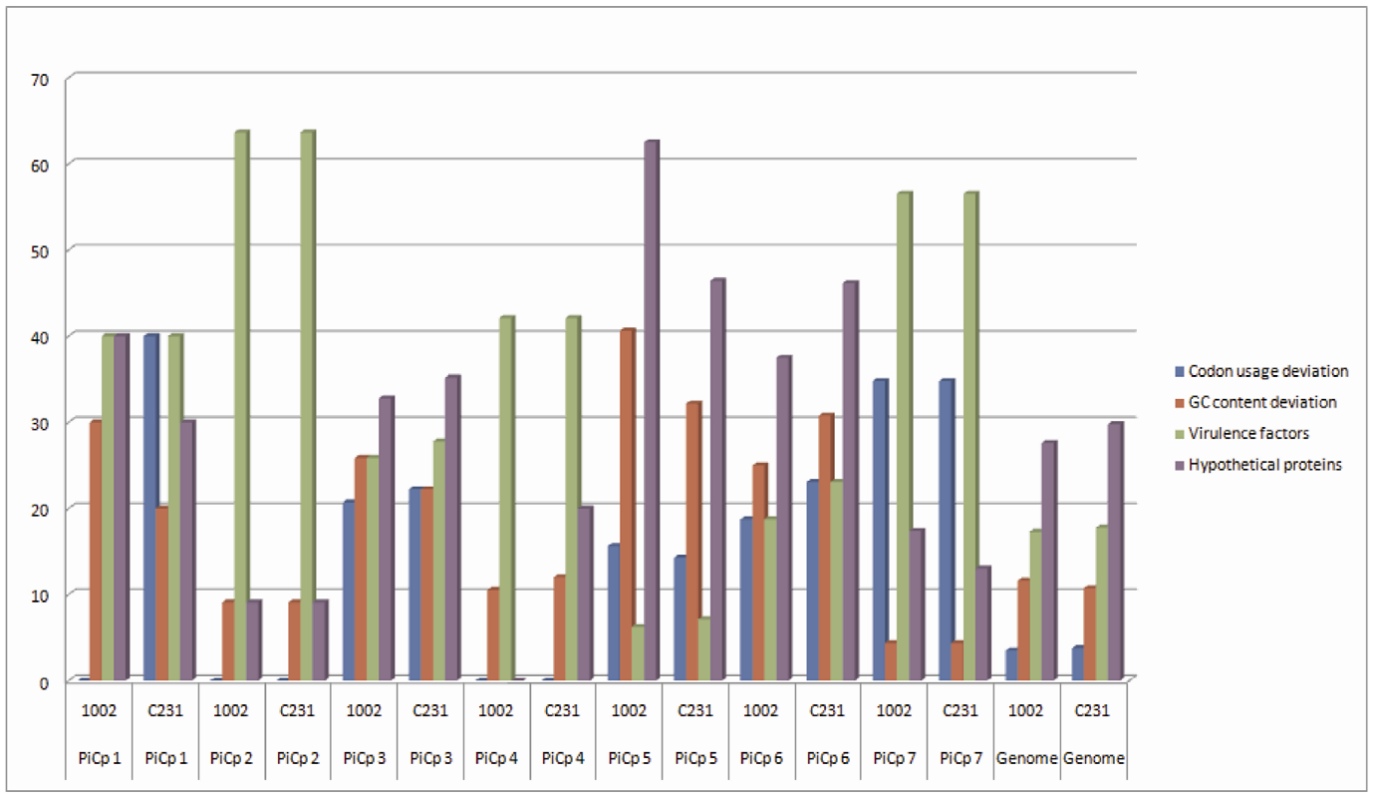
Frequencies of PAI features within the PICPs and in the full genomes of *C. pseudotuberculosis* strains 1002 and C231. Y-axis: frequency in percentage; X-axis: PICPs and genomes of *C. pseudotuberculosis* strains 1002 and C231. The frequencies of the features in each PICP and in the whole genomes of the two strains are represented in the following colors: blue for codon usage deviation; red for GC content deviation; green for virulence factors; and purple for hypothetical proteins.

In a comparison of the frequency of CDSs with codon usage deviation, *C. pseudotuberculosis* PICPs 3, 5, 6 and 7 had higher frequencies than those found in the whole genome of *C. pseudotuberculosis* 1002. In *C. pseudotuberculosis* C231, together with the previously described PAIs (PICPs 3, 5, 6 and 7), *C. pseudotuberculosis* PICP1 also had a greater frequency of CDSs with codon usage deviation than that of the whole genome (Figure 3). This observation may mean that *C. pseudotuberculosis* PICP1 has become more adapted to the acceptor's codon usage in strain 1002 when compared to the same PAI in strain C231. The frequency of CDSs with G+C content deviation in strains 1002 and C231 was higher in *C. pseudotuberculosis* PICPs 1, 3, 5 and 6 (Figure 3).

In general, the frequency of genes with similarity to virulence factors in PAIs was greater than that in the rest of the genome, except for *C. pseudotuberculosis* PICP5. However, this island, along with *C. pseudotuberculosis* PICPs 3 and 6, had higher frequencies of hypothetical proteins.

No single characteristic was consistent throughout all *C. pseudotuberculosis* PICPs. However, the absence of *C. pseudotuberculosis* PICPs in non-pathogenic bacteria, in addition to a high frequency of at least one of the classic PAI features, and the finding of virulence genes were used as determining factors for the characterization of a PAI.

### Co-occurrence of pathogenicity islands in *C. pseudotuberculosis* and *C. diphtheriae*

*C. pseudotuberculosis* PICPs were compared to the genome of *C. diphtheriae* NCTC 13129 to determine whether these islands are present in this organism.

**Figure 4:**
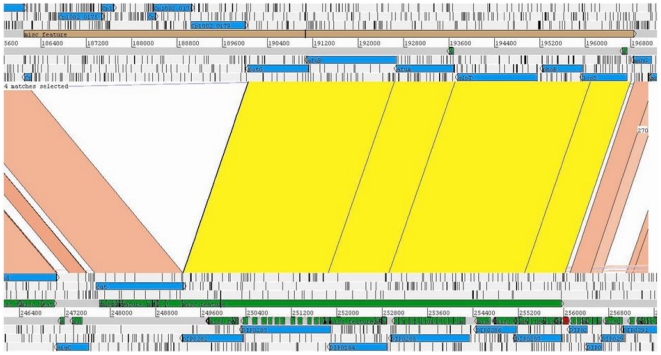
PICP3 and PICD3 (top and bottom, respectively) in the *C. pseudotuberculosis* and *C. diphtheriae* genomes. Cp1002 and *C. diphtheriae* NCTC 13129 are shown at the top and bottom, respectively. Regions of similarity between the two genomes are marked in pink. Regions of similarity between two PAIs are marked in yellow, showing the presence of PICD3 in *C. pseudotuberculosis* with an insertion. Image generated by ACT (the Artemis Comparison Tool).

Interestingly, most *C. pseudotuberculosis* PICP3 genes are found in the genome of *C. diphtheriae* NCTC 13129, with the same gene order, identified as *C. diphtheriae* PICD 3 (Figure 4). The presence of this PAI in two pathogenic species and its absence in non-pathogenic *C. glutamicum* provide evidence for the importance of this region for determining the virulence of *C. pseudotuberculosis* and *C. diphtheriae*.

**Figure 5:**
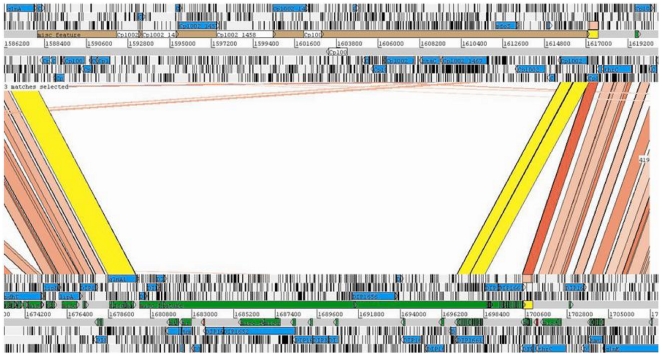
Replacement of the *C. diphtheriae* PICD8 (bottom) with *C. pseudotuberculosis* PICP5 (top). Regions of similarity are represented by lines between the two genomes. The flanking regions of PICD8 and PICP5 are highlighted in yellow, showing the region of replacement. Image generated by ACT (the Artemis Comparison Tool).

Moreover, the flanking regions of the PICP5 of *C. pseudotuberculosis* are the same as those of PICD8 of *C. diphtheriae* (Figure 5). This pattern highlights this region as a putative “hotspot” for the insertion of transposons and, most likely, GEIs.

### Conclusions

Pathogenicity islands play a major role in the virulence of pathogenic bacteria, and therefore, their correct identification and characterization may provide valuable data.

We developed software (PIPS) that accurately identifies pathogenicity islands; it is easy to install, which makes it accessible even to researchers with little computational knowledge. In addition, this software has a web-based interface that is platform and installation independent, facilitating fast analysis. Moreover, PIPS uses a complete approach that is based on the detection of multiple PAIs, i.e., atypical G+C content, codon usage deviation, virulence factors, hypothetical proteins, transposases, flanking tRNA and its absence in non-pathogenic organisms.

During the validation, this software identified 12 of the 13 previously described *C. diphtheriae* PAIs, demonstrating its superior efficiency compared to the other currently available software systems, which identified 6 and 10 PAIs (PredictBias and IslandViewer, respectively). Furthermore, PIPS achieved a high overall sensitivity, specificity and accuracy in identifying PAIs in *C. diphtheriae* NCTC13129 and *E. coli* CFT073. Moreover, we predicted 7 PAIs in *C. pseudotuberculosis* and showed that no single characteristic was consistent throughout all of the *C. pseudotuberculosis* PICPs. This latter finding, in addition to our success with this program, highlights the need for a multi-pronged strategy toward PAI identification that heavily weights the absence in a closely related non-pathogenic organism in addition to signs of HGT and the presence of virulence factors.

Finally, the identification of *C. pseudotuberculosis* PICP3, an island that is shared by *C. pseudotuberculosis* and *C. diphtheriae*, along with the identification of *C. pseudotuberculosis* PICP5, an island that is located in a putative “hotspot”, corroborates the accuracy of the program for correct identification of PAIs.

Future PIPS development will focus on increasing the software speed in searches for insertion sequences. The next versions will also aim to facilitate analysis through the implementation of a graphic interface and minimization of the required programs (Availability and requirements are described in Appendix S1).

## Supporting Information

Figure S1 **Prediction of PICD12 of** ***C. diphtheriae*** **with a different size than the literature prediction.** At the top, the *C. diphtheriae* genome; at the bottom, the *C. glutamicum* genome. In green, highlighted by an orange box, *C. diphtheriae* PICD12 as described in the literature; in red, an additional region identified by PIPS. This image was generated by ACT.
(DOC)

Figure S2 **Graphic representation of PAI features in the genome (A) and in the pathogenicity islands (B) of C. pseudotuberculosis and C. diphtheriae.** Y-axis: frequency as a percentage; X-axis: codon usage deviation, GC content deviation, virulence factors and hypothetical proteins. C. diphtheriae strain NCTC 13129 is in blue, and C. pseudotuberculosis strains 1002 and C231 are in red and green, respectively. (A) Frequency of the PAI features in the genomes and (B) frequency of the PAI features in the pathogenicity islands of the bacteria.
(DOC)

Table S1 **PAI composition.** The PAIs composition of the *C. diphtheriae* strain NCTC 13129, as described in the literature and as identified by PIPS, IslandViewer and PredicBias.
(DOC)

Appendix S1 **Availability and Requirements.**
(DOC)
