# Supplementary figures and images for: PIPS: Pathogenicity Island Prediction Software
Source: PLoS One. 2012 Feb 15;7(2):e30848. doi: 10.1371/journal.pone.0030848 (PMC3280268; doi:10.1371/journal.pone.0030848)

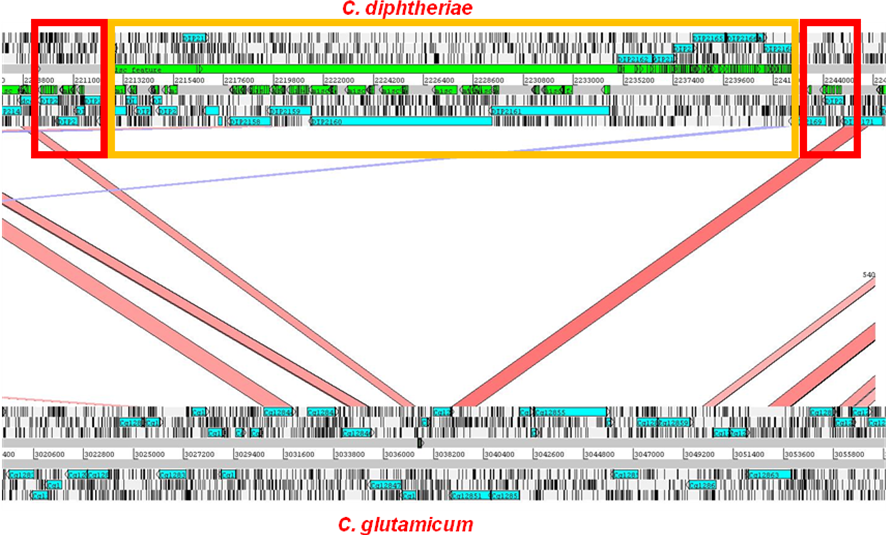

Supplement: Figure S1 — Prediction of PICD12 of C. diphtheriae with a different size than the literature prediction. At the top, the C. diphtheriae genome; at the bottom, the C. glutamicum genome. In green, highlighted by an orange box, C. diphtheriae PICD12 as described in the literature; in red, an additional region identified by PIPS. This image was generated by ACT. (DOC) [file pone.0030848.s001.doc]

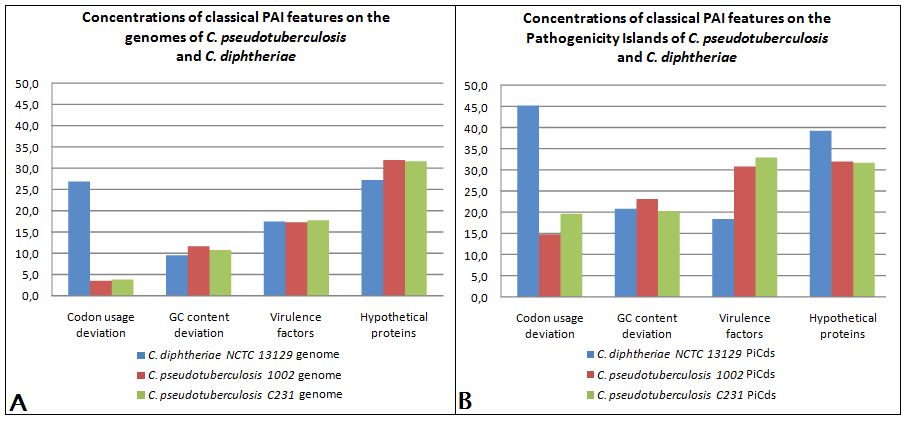

Supplement: Figure S2 — Graphic representation of PAI features in the genome (A) and in the pathogenicity islands (B) of C. pseudotuberculosis and C. diphtheriae. Y-axis: frequency as a percentage; X-axis: codon usage deviation, GC content deviation, virulence factors and hypothetical proteins. C. diphtheriae strain NCTC 13129 is in blue, and C. pseudotuberculosis strains 1002 and C231 are in red and green, respectively. (A) Frequency of the PAI features in the genomes and (B) frequency of the PAI features in the pathogenicity islands of the bacteria. (DOC) [file pone.0030848.s002.doc]
