# Supplementary material for: PIPS: Pathogenicity Island Prediction Software
Source: PLoS One. 2012 Feb 15;7(2):e30848. doi: 10.1371/journal.pone.0030848 (PMC3280268; doi:10.1371/journal.pone.0030848)
Supplement: Table S1 — PAI composition. The PAIs composition of the C. diphtheriae strain NCTC 13129, as described in the literature and as identified by PIPS, IslandViewer and PredicBias. (DOC) [file pone.0030848.s003.doc]

| **PAI** | **Literature** | **PIPS** | **IslandViewer** | | | **PredictBias** | **Classification attibutted by PredictBias** |
| --- | --- | --- | --- | --- | --- | --- | --- |
|  |  |  | **Colombo SIGI-HMM** | **IslandPath DIMOB** | **IslandPick** |  |  |
| PiCd 1 | DIP0180-DIP0222 | DIP0179-DIP0222 | DIP0177-DIP0179  DIP0219-DIP0222 | Not identified | DIP0180-DIP0222 | DIP0183-DIP0207 | GEI |
| PiCd 2 | DIP0223-DIP0244 | DIP0223-DIP0247 | DIP0223-DIP0226  DIP0242-DIP0250 | Not identified | DIP0227-DIP0241 | DIP0226-DIP0235 | GEI |
| PiCd 3 | DIP0282-DIP0287 | DIP0282-DIP0290 | DIP0281-DIP0290 | Not identified | Not identified | DIP0279-DIP0289 | PAI |
| PiCd 4 | DIP0334-DIP0357 | DIP0334-DIP0359 | Not identified | Not identified | Not identified | DIP0333-DIP0339 | GEI |
| PiCd 5 | DIP0438-DIP0445 | Not identified | Not identified | Not identified | Not identified | Not identified | - |
| PiCd 6 | DIP0752-DIP0766 | DIP0750-DIP0766 | Not identified | Not identified | DIP0752-DIP0766 | Not identified | - |
| PiCd 7 | DIP0795-DIP0820 | DIP0794-DIP0823 | DIP0806-DIP0821 | DIP0807-DIP0822 | Not identified | DIP0795-DIP0804 | GEI |
| PiCd 8 | DIP1645-DIP1663 | DIP1645-DIP1664 | Not identified | Not identified | Not identified | Not identified | - |
| PiCd 9 | DIP1817-DIP1837 | DIP1817-DIP1843 | Not identified | DIP1817-DIP1841 | DIP1817-DIP1837 | Not identified | PAI |
| PiCd 10 | DIP2010-DIP2015 | DIP2010-DIP2015 | DIP2010-2015 | Not identified | Not identified | Not identified | - |
| PiCd 11 | DIP2066-DIP2093 | DIP2064-DIP2093 | Not identified | Not identified | DIP2063-DIP2081  DIP2083-DIP2094 | Not identified | - |
| PiCd 12 | DIP2148-DIP2168 | DIP2143-DIP2170 | Not identified | Not identified | DIP2143-DIP2167 | Not identified | - |
| PiCd 13 | DIP2208-DIP2234 | DIP2208-DIP2234 | Not identified | Not identified | DIP2207-DIP2227 | DIP2208-DIP2217 | GEI |
