# Supplementary material for: PIPS: Pathogenicity Island Prediction Software
Source: PLoS One. 2012 Feb 15;7(2):e30848. doi: 10.1371/journal.pone.0030848 (PMC3280268; doi:10.1371/journal.pone.0030848)
Supplement: Appendix S1 — Availability and Requirements. (DOC) [file pone.0030848.s004.doc]

# Availability and requirements

- **Project name:** PIPS
- **Project home page:** <http://www.genoma.ufpa.br/lgcm/pips>
- **Link on bioinformatics.org**: <http://www.bioinformatics.org/groups/?group_id=1063>
- **Operating system(s):** UNIX Platform
- **Programming language:** Perl
- **Other requirements:** Java Virtual Machine v1.6.0_20, HMMER3, PERL v5.10.1, COLOMBO/SIGI-HMM v3.8 or higher
- **License:** GNU GPL
- **Restrictions for use by non-academics:** None
- **Run time:** Varies from 20 min to 1 h for 2.48-5.23 Mb genomes using a computer with two 2.20 GHz processors and 4 GB of RAM.
